# Evaluating the Impact of an mHealth Platform for Managing Acute Postoperative Dental Pain: Randomized Controlled Trial

**DOI:** 10.2196/49677

**Published:** 2023-10-20

**Authors:** Bunmi Tokede, Alfa Yansane, Ana Ibarra-Noriega, Joanna Mullins, Kristen Simmons, Nicholas Skourtes, Urvi Mehta, Sayali Tungare, David Holmes, Joel White, Muhammad Walji, Elsbeth Kalenderian

**Affiliations:** 1 The University of Texas Health Science Center, Houston, TX, United States; 2 School of Dentistry, University of California San Francisco, San Francisco, CA, United States; 3 Willamette Dental Group, Hillsboro, OR, United States; 4 FollowApp.Care, London, United Kingdom; 5 Harvard School of Dental Medicine, Boston, MA, United States

**Keywords:** mobile health, patient-reported outcomes, acute pain, dentistry, dental, dentist, pain, mHealth, patient-reported outcome, PRO, patient-reported outcome measures, PROs, PROM, randomized controlled trial, RCT

## Abstract

**Background:**

Postoperative dental pain is pervasive and can affect a patient’s quality of life. Adopting a patient-centric approach to pain management involves having contemporaneous information about the patient’s experience of pain and using it to personalize care.

**Objective:**

In this study, we evaluated the use of a mobile health (mHealth) platform to collect pain-related patient-reported outcomes over 7 days after the patients underwent pain-inducing dental procedures; we then relayed the information to the dentist and determined its impact on the patient’s pain experience.

**Methods:**

The study used a cluster-randomized experimental study design with an intervention arm where patients were prompted to complete a series of questions relating to their pain experience after receiving automated text notifications on their smartphone on days 1, 3, 5, and 7, with the resulting information fed back to dentists, and a control arm where patients received usual care. Providers were randomized, and patients subsequently assumed the enrollment status of their providers. Providers or their staff identified eligible patients and invited them to participate in the study. Provider interviews and surveys were conducted to evaluate acceptance of the mHealth platform.

**Results:**

A total of 42 providers and 1525 patients participated. For the primary outcome (pain intensity on a 1 to 10 scale, with 10 being the most painful), intervention group patients reported an average pain intensity of 4.8 (SD 2.6), while those in the control group reported an average pain intensity of 4.7 (SD 2.8). These differences were not significant. There were also no significant differences in secondary outcomes, including pain interference with activity or sleep, patient satisfaction with pain management, or opioid prescribing. Patient surveys revealed reluctance to use the app was mostly due to technological challenges, data privacy concerns, and a preference for phone calls over texting. Providers had high satisfaction with the app and suggested integrating additional features, such as an in-system camera for patients to upload pictures and videos of the procedural site, and integration with the electronic health record system.

**Conclusions:**

While the mHealth platform did not have a significant impact on acute postoperative pain experience, patients and providers indicated improvement in patient-provider communication, patient-provider relationship, postoperative complication management, and ability to manage pain medication prescribing. Expanded collaboration between mHealth developers and frontline health care providers can facilitate the applicability of these platforms, further help improve its integration with the normal clinic workflow, and assist in moving toward a more patient-centric approach to pain management.

## Introduction

The experience of pain is a national and global public health problem with significant physical, cognitive, and emotional costs [1-3]. Postoperative dental pain, in particular, is pervasive and can significantly affect a patient’s quality of life and ability to perform daily activities [4]. Untreated or poorly managed postoperative dental pain can lead to complications such as infection, delayed healing, and the need for additional dental treatment [5]. Effective pain management should prioritize the individual needs and preferences of each patient. This patient-centered approach may involve tailoring the treatment plan to the patient’s specific needs, providing clear and concise information about pain management options, and actively involving the patient in the decision-making process. By taking a more holistic approach to acute pain management, health care professionals can help ensure that patients receive the most effective and personalized care possible [6].

While dentists are prescribing fewer postoperative opioids [7], current practice suggests that opioid prescriptions are often discordant with evidence-based prescription guidelines [8], especially after common oral surgery procedures such as dental extractions. Third molar extractions are the dental procedures most likely to be associated with an opioid prescription [9]. This is problematic because dentists are responsible for a disproportional share of opioids prescribed to adolescents, for whom even a single opioid prescription increases the lifetime risk of future opioid abuse [10]. One reason for the inappropriate prescribing of opioids is that oral health providers are unable to accurately predict or actively monitor postoperative pain. Their desire to prevent unwanted unscheduled visits leads them to pre-emptively prescribe opioids in an attempt to satisfy patients’ short-term pain management expectations. Patient expectations of receiving the most effective pain relievers, coupled with diminished patient satisfaction and negative reviews if their expectations are not met, provide yet another perverse incentive for pre-emptive opioid prescriptions [11].

Adopting a patient-centric approach to pain management involves collecting valuable information about the patient’s experience of pain and related factors, and providing the information back to the dentist to help manage the care [12]. Patient-reported outcomes (PROs) and PRO measures play a crucial role in this process. PROs refer to any report of the patient’s health status that comes directly from the patient, while patient-reported outcome measures are validated questionnaires that patients complete to self-assess their health status [13]. Patient self-reporting is a critical part of comprehensive pain assessment [14], given pain’s subjective and multidimensional nature. PROs allow clinicians to directly assess patients’ symptoms, symptom burden, functional status, health behaviors, health-related quality of life, and care experiences [15], and deliver value-based care.

Against this backdrop, the use of mobile health (mHealth) systems for the collection of PROs is on the rise [16-21]. An mHealth system is a platform that incorporates mobile devices, wireless communication technologies, and software apps to deliver health care services and information to patients and health care providers. These platforms can be designed for various purposes such as remote monitoring of patients, disease management, or telemedicine and are potentially powerful platforms for the delivery of behavior change interventions because they can improve engagement with established strategies for prevention and treatment through personalized goal setting, individualized dosing reminders, and gamification [22]. By leveraging mHealth systems to collect, integrate, and analyze PROs, providers can efficiently gather valuable information about the patient’s pain experience and improve the effectiveness of pain management strategies. In dentistry, the timely and efficient capture of PRO data, such as postoperative pain experience, through an mHealth system is lacking, and this represents a missed opportunity to improve patient outcomes, care experience, and provider performance.

Therefore, this study aimed to assess the impact of an mHealth platform on acute dental postoperative pain management in terms of pain experience and patient satisfaction. We also explored the providers’ perspectives on the use of mobile technology in the management of acute postoperative dental pain.

## Methods

### Study Overview

A 24-month phase 2 cluster randomized controlled trial was conducted to evaluate the impact of using an mHealth platform on patient postoperative pain experiences, satisfaction with pain management, and dental provider satisfaction with the platform. The multicenter study was conducted at an academic dental institution and a large privately held dental group practice. Data collection spanned February 2020 through January 2022. Consented providers or staff identified eligible patients and invited them to participate in the study.

### Study Sites and Participants

The study was conducted at two dental institutions. One is part of an academic dental site and the other is a large privately held dental group practice of around 50 offices across the Pacific Northwest region of the United States. The academic dental center comprises predoctoral, resident, and faculty clinics. The patient population provides a diverse sample in terms of demographics and socioeconomic status.

The provider inclusion criteria were being a general dentist or specialist in oral and maxillofacial surgery endodontics, or periodontics; performing any one or combination of the identified potentially pain-inducing procedures (see list below); practicing for a minimum of two clinic sessions per week (ie‚ one full clinic day); having a minimum of 6 months of practice experience; and having access to and willingness to use a smartphone.

The patient inclusion criteria were being 18 years or older and having access to and ability to use a smartphone.

### Included “Pain-Inducing” Procedures

The core set of pain-associated dental procedure codes (Code to Dental Terminology; American Dental Association) included were endodontics: D3310, D3320, D3330, D3346, D3347, D3348, D3410, D3421, D3425, D3426, and D3450; periodontal surgery: D4210, D4211, D4212, D4240, D4241, D4249, D4260, D4261, and D4263; oral surgery: D7210, D7220, D7230, D7240, D7241, D7250, D7310, D7311, D7320, and D7321; and implant dentistry: D6010, D6011, D6012, D6013, D6040, D6050, D6100, D6101, D6102, D6103, D6104, and D6081.

### Intervention

The mHealth platform deployed in this study was FollowApp.Care. A detailed description of the platform has been previously published [23]. Briefly, FollowApp.Care is a communications platform to collect patient-generated health data before or after a procedure. The platform is designed to inform treatment decisions, improve patient care, and generate performance reports. FollowApp.Care can be accessed through any SMS text message–enabled smartphone.

On completion of any of the eligible procedures, enrolled patients (including intervention and control groups) received postoperative care instructions and guidance according to each institution’s standard practice (usual care). The intervention group received additional guidance about FollowApp.Care, including the timing and frequency of text notifications and when to expect a response from providers or office staff, if necessary. Patients in the intervention group received text notifications at predetermined time intervals (eg, 9 AM) on days 1, 3, 5, and 7 prompting them to complete a brief pain assessment survey covering the preceding 24-hour period. Additionally, a comment/chat feature enabled patients to securely communicate more information to their dental care team through FollowApp.Care when needed. The control group received usual care and was advised to contact their providers or dental offices through the usual channels if they experienced any unexpected symptoms or had any complaints or questions. Control participants filled out the PRO pain survey only on day 7. To ensure that FollowApp.Care was implemented as intended, fidelity was also measured.

### Randomization

**Figure 1.**
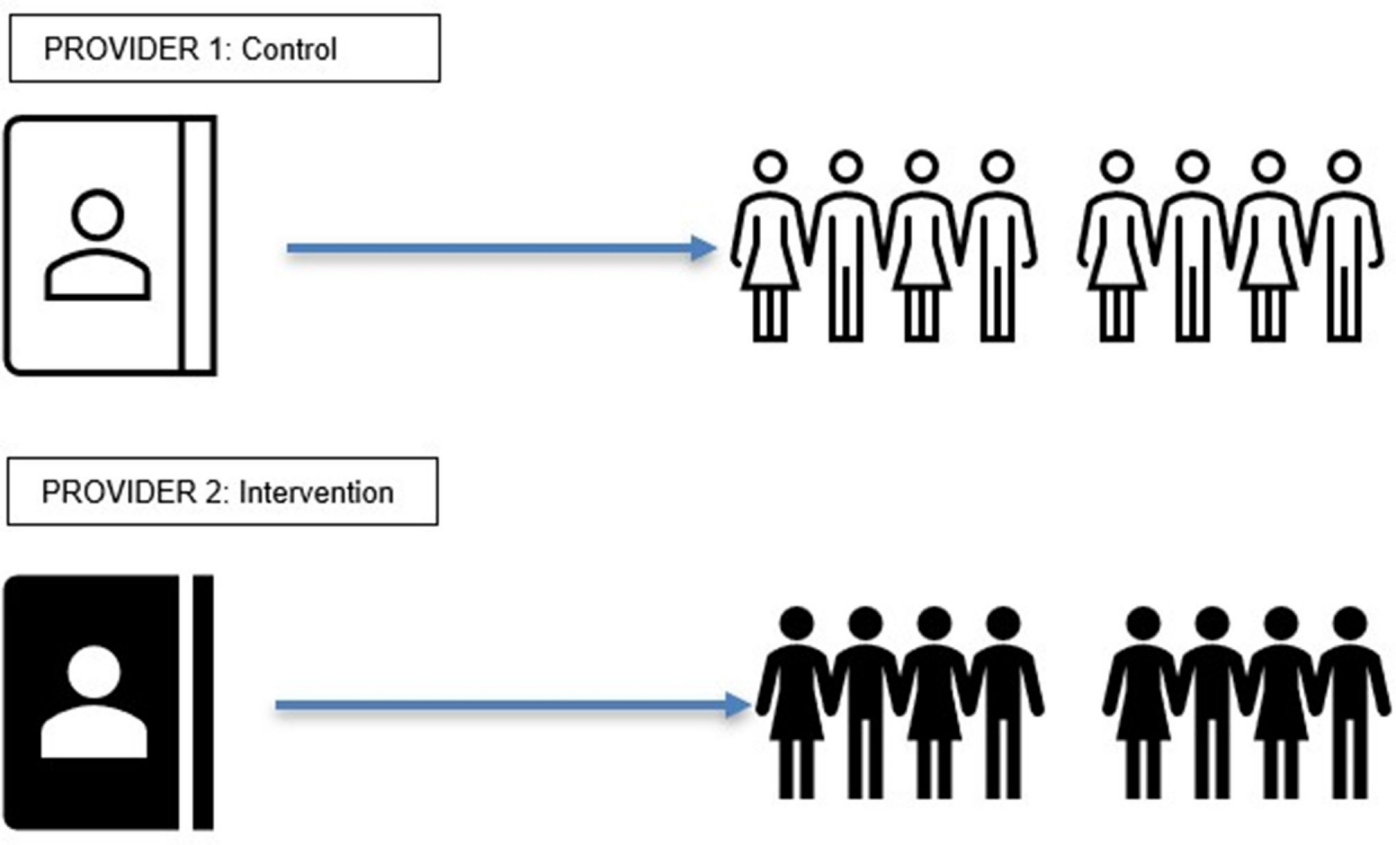
Randomization scheme: each provider represents a single cluster that was randomized to either the intervention or control group. Each participating patient seen would then assume the randomization status of their provider.

Each of the participating providers was randomized to one study arm (the mHealth intervention plus standard care vs standard care only), and each patient automatically assumed the randomization status of their provider. As such, each patient was nested within a specific provider (Figure 1). Randomization was conducted using pseudorandom number generation, and randomization codes were maintained by the statistician in a secure cloud-based storage system. Each week, consenting providers or their staff identified eligible patients and invited them to participate in the study. Using a standardized template provided by the research team, clinic staff members (who had undergone training in human subjects’ protection) obtained informed consent from interested patients to confirm their willingness to participate in the study before their procedures. The intervention could not be masked.

### Means of Data Collection

We used the mHealth platform (FollowApp.Care) to collect PRO data (pain experience) from patients after dental procedures. Electronic health record (EHR) data for postprocedure prescribing data was extracted using the patient enrollment data. EHR data was then merged with the mHealth survey response data for each patient.

### Study Outcomes

The primary PRO of interest was pain intensity—an assessment of the worst severity of pain experienced during the 7 days after an eligible dental procedure—and the data was collected using an item from the validated Patient-Reported Outcomes Measurement Information System (PROMIS) Shortform 3A Version 1 questionnaire [24]. The response categories range from “No pain” to “Very severe” and are measured on a 0 to 10 rating scale. The outcome was treated as continuous.

### Secondary Outcomes

#### Pain Interference

Pain interference, defined as interference with activity (walking, work, general activity, sleep) and interference with affect (mood, enjoyment of life), was captured using 3 items taken from the validated Revised American Pain Society Patient Outcome Questionnaire (APS-POQ-R) form [25]. Response categories ranged from “No interference” to “High interference” on a 0 to 10 rating scale. Each item queries how, in the last 7 days, pain interfered with doing activities such as walking, sitting in a chair, or standing at the sink; falling asleep; and staying asleep.

#### Patient Satisfaction

Satisfaction with how pain was managed was assessed with the following two statements from the validated APS-POQ-R form, which was measured on a 0 to 10 rating scale [25]:

1. Ability to participate in decisions about pain treatment
2. Satisfaction with the results of your pain treatment

#### Use of Opioid Medications

The proportion of participating patients who got a postoperative opioid prescription was assessed using data from the patient EHR. Through secondary analysis of the EHR, medication-prescribing patterns were collected by deploying query scripts to identify the patients who received the prescriptions postoperatively, including type, dosage, frequency, and duration.

### Sample Size

Among the 2 included dental sites, a total of 42 providers were recruited to participate in the study over the 2-year study period. Each provider was expected to reasonably recruit 19 patients per year. The expected number of patients was 1596. Adjusting for a 60% response rate among recruited patients, we calculated a sample of 958. Given a total sample size of 958 patients, a standard significance level of .05 (α=.05), and a within-cluster correlation coefficient of 0.1 (ρ=0.1), we estimated that the power to detect a 2.0-unit effect difference in pain would be 80.7%. The minimum power achieved was derived using R (version 4.3.1 for Windows; R Foundation for Statistical Computing; longpower package). All statistical analyses were performed at the standard significance level (α=.05) using R.

### Statistical Methods

Means and corresponding estimates of precision (eg, SDs and 95% CIs) and frequency distributions with percentage contributions were used to report the distribution of each variable included in the quantitative analyses. To test whether there was a difference in pain intensity, interference, or satisfaction with pain management between the study groups on day 7, a hierarchical model was performed that adjusted for within-clinic correlations and repeated measures over patient responses. Models included the procedure type, time, age, gender, and race/ethnicity.

### Fidelity

Fidelity was measured using metrics as outlined in Textbox 1.

Textbox 1. **Fidelity metrics for patients and providers.**

**Patient fidelity measures**

- Provided verbal consent and received the information sheet.
- FollowApp.Care profile was created
- Received text notifications on day 0
- Patient response time
- Number of patients who have phone service provided by T-Mobile
- Response rate day 1
- Response rate day 3
- Response rate day 5
- Response rate day 7

**Dentist fidelity measures**

- Signed consent forms before training
- Completed 1-hr training
- Verified FollowApp.Care profile
- Unique identifiers provided
- Completed Unified Theory of Acceptance and Use of Technology survey
- Number of log-ins
- Number of successful log-ins
- Number of unsuccessful log-ins
- Number of alerts triggered
- Number of alerts resolved
- Number of alerts resolved by chat
- Number of alerts resolved by phone
- Number of alerts resolved by acknowledgment
- Number of alerts unresolved
- Average response time to alerts

### Assessing Provider Acceptance

To assess whether practitioners were unduly burdened by the technology and whether it fit seamlessly into their workflow, the Unified Theory of Acceptance and Use of Technology (UTAUT) questionnaire was administered to those in the intervention group. Four key constructs were measured: performance expectancy, effort expectancy, social influence, and facilitating conditions. A descriptive analysis was performed to describe the constructs of the UTAUT questionnaire.

Semistructured virtual interviews were also conducted with dental care providers from both study sites. Nine of these interviews were with dentists alone, and five were group interview sessions with dental care teams that consisted of dentists, dental assistants, dental hygienists, or dental clinic administrative staff. The main aim of these interviews was to evaluate the provider’s experience with using the mHealth platform for managing their patients’ postoperative pain, including its impact on their clinic workload, workflow patterns, and satisfaction with the effectiveness of pain management. Our analysis for this research focused on using this interview data to identify the barriers and facilitators for using an mHealth platform for postoperative acute pain management and communication. One trained interviewer conducted all the interviews. Each interview was audio or video recorded through Zoom video telephonic software (Zoom Video Communications) and then transcribed using Rev speech-to-text transcription services. For the qualitative analysis, a combination of deductive and inductive approaches was used. Independent coding of the transcripts was performed by 2 of the authors, the coding was assessed and discussed for variation and consensus, and codes were identified that fit into the predefined themes of barriers and facilitators for the use of the mHealth platform. The framework method of analysis [26] was used to organize and analyze the codes and themes.

### Ethical Considerations

The study protocol was reviewed and approved by the University of Texas Institutional Review Board (IRB# 18-25477) and registered on ClinicalTrials.gov (NCT03881891). Using a standardized template provided by the research team, providers or clinic staff members obtained informed consent from interested patients before their respective surgical procedures.

## Results

### Patient and Provider Population

A total of 42 providers (intervention: n=24; control: n=18), consisting of 24 general dentists, 16 endodontists, and 2 oral surgeons, participated in the trial. The study included 1525 patients (intervention: n=851; control: n=674) with an average age of 44.5 (SD 14.3) years, of whom 675 (44.3%) were female and 865 (56.7%) were White (Table 1). The most common procedures were oral surgery procedures.

**Table 1.** Patient Characteristics.

Variable		Control (n=674)		Intervention (n=851)	
**Gender, n (%)**					
	Male	255 (37.8)		292 (34.3)	
	Female	313 (46.4)		362 (42.5)	
	Other	106 (15.8)		197 (23.2)	
**Race n (%)**					
	Asian	7 (1.0)		19 (2.2)	
	American Indian/Alaska Native	3 (0.4)		0 (0.0)	
	Black	5 (0.7)		9 (1.1)	
	Hispanic/Latino	35 (5.2)		45 (5.3)	
	Native Hawaiian/Pacific Islander	5 (0.7)		4 (0.5)	
	White	432 (64.1)		433 (50.9)	
	More than one race	18 (2.7)		29 (3.4)	
	Other	5 (0.7)		4 (0.5)	
	Unknown	164 (24.3)		308 (36.2)	
Age of patients (years), mean (SD)		44.8 (13.8)		44.3 (14.6)	

### Fidelity

Response rates for the mHealth-administered surveys were 56.9% (484/851) on day 1 and 49.8% (424/851) on day 7 for intervention patients, and 42% (283/674) for control patients. All patients had a FollowApp.Care profile created, and 98.3% (1504/1525) received the day 1 SMS text message notification, with an average response time of about 7 hours. Over the study duration, 349 alerts were generated. Of these, 335 were resolved, 14 were unresolved, and the average response time to patient alerts was 8 hours and 48 minutes.

### Postoperative Pain Experience

For the primary outcome “How intense was your pain at its worst following your procedure?” (pain intensity), intervention group patients reported an average pain intensity of 4.8 (SD 2.6), while those in the control group reported an average pain intensity of 4.7 (SD 2.8). These differences were not significant. Intervention group patients also responded to the following question: “What is your pain level right now?” The mean pain intensity ranged from 2.9 (SD 2.4) on day 1 to 1.2 (SD 1.8) on day 7 post procedure.

Table 2 shows that there were no significant differences in interference in falling asleep and staying asleep between the intervention and control groups.

**Table 2.** Pain interference scores for activities, falling asleep, and staying asleep.

	Pain interference score		*P* value
	Control (n=674)	Intervention (n=851)	
Activities	0.8	1	.19
Falling asleep	1.6	1.9	.08
Staying asleep	1.4	1.7	.36

### Patient Satisfaction

In response to the question “Were you allowed to participate in decisions about your pain treatment as much as you wanted to? (0, least to 10, most),” respondents in the intervention group reported an average of 7.7 (SD 3.5) out of 10 in participation in decision-making, while those in the control group reported an average of 8.3 (SD 3.0). When asked “Select the one number that best shows how satisfied you are with the results of your pain treatment,” respondents in the intervention group reported an average of 8.6 (SD 2.2) out of 10, while those in the control group reported 8.9 (SD 2.0). There was no significant difference between the groups.

### Use of Opioid Medications

**Figure 2.**
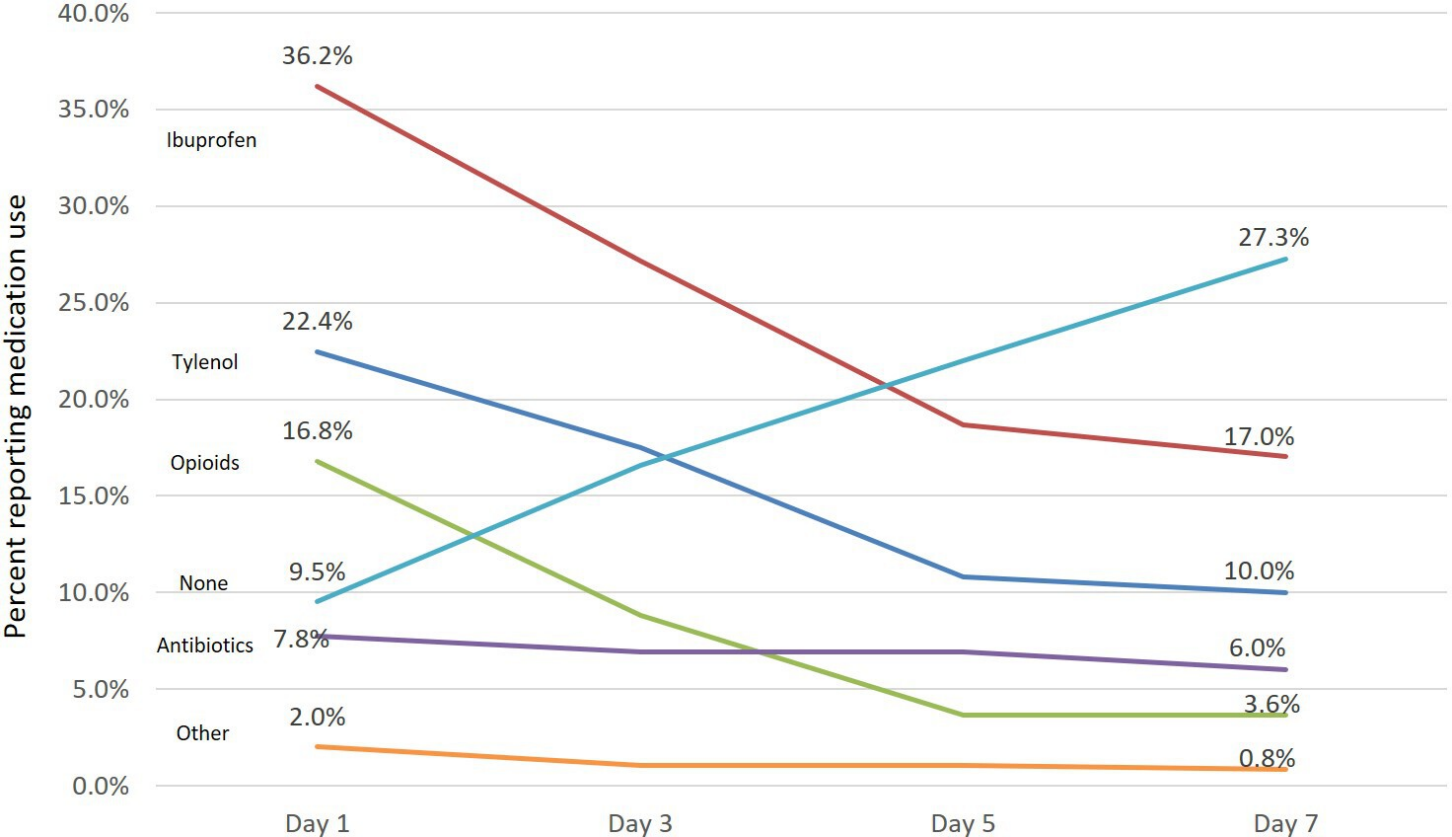
Patient-reported medication use.

Figure 2 displays the most frequently used patient-reported medications. Descriptive statistics were used to determine the distribution of opioids prescribed to the responding patients by the providers. In total, 26.4% (225/851) of patients in the intervention group were prescribed an opioid, while 16.8% (113/674) of those in the control group were prescribed an opioid. Nearly 50% of the opioid prescriptions were written by only 3 providers. Using the mHealth platform did not appear to have an effect on the odds of opioids prescribed after a dental procedure (odds ratio 1.17, 95% CI 0.61-1.64; *P*=.40) after adjusting for gender, procedure group, and provider.

### Adjusted Analysis

The regression analysis showed that there was no statistically significant difference between the intervention and control arms for all study outcomes, after adjusting for provider, gender, and procedure type.

### Provider Experience With the mHealth App

Results from the UTAUT questionnaire indicated that most providers found the platform useful, clear, and understandable; that their organization in general thought they should use it; and that they have the necessary resources and knowledge to use the platform. The validated UTAUT questionnaire was administered to 18 intervention providers. The four UTAUT constructs are associated with a behavioral intention to use the new technology (FollowApp.Care app); high scores on each of the constructs are associated with a higher behavioral intention to use the FollowApp.Care platform. The responses to the four items that form the performance expectancy construct showed that most providers found FollowApp.Care useful, enabling them to perform tasks more quickly, increasing productivity, and increasing the chances of a positive performance review. Median scores for each item were ≥4 on a 7-point Likert scale. The responses to the four items that form the effort expectancy construct showed that most providers found that FollowApp.Care is clear and understandable, believing that they can become skillful and that the platform is easy to use and operate. Median scores for each item were ≥5 on a 7-point Likert scale. The responses to the four items that form the social influence construct showed that most providers found that those who influence their behavior, people who are important to them, and their clinical management as well as the organization in general thought that they should use the platform. Median scores for each item were ≥5 on a 7-point Likert scale. The responses to the four items that form the facilitating conditions construct show that most providers found that they have the necessary resources and knowledge to use FollowApp.Care, that it is generally compatible with other systems that they use, and that there is assistance for its operation. Median scores for each item were ≥4 on a 7-point Likert scale (see Multimedia Appendix 1 for descriptive tables).

### Qualitative Analyses

Three main themes were identified from the perspective of providers regarding the use of the platform for postoperative acute pain management: (1) potential facilitators and barriers to adoption, (2) patient acceptance and hesitancy, and (3) future use of the platform (Multimedia Appendix 2). Providers seemed to appreciate the improved accessibility for patients.

*It seemed like we were a lot more accessible. It would lower their anxiety or if they were scared that something was going on, they were able to get answers a lot quicker. I think most of the patients liked it from what I remember hearing*.

The chat feature was particularly helpful in facilitating direct communication between dentists and patients, resulting in improved patient care and stronger patient-dentist relationships. Dentists found the alert system useful for identifying patients with specific symptoms and reducing unnecessary postoperative appointments. However, they also reported feeling a lack of personal touch through mHealth SMS text messaging. Furthermore, they thought it represented an additional burden on their workload and an invasion of their time due to receiving messages and alerts after work hours.

A*ctually sometimes it (using the platform) was just an additional step, because I had to respond on the app and then call the patient because they still needed to talk to me. I still needed to talk to them. I felt like texting them wasn’t enough. You know? So, yeah, I think in that way it was probably good for data collection and everything, but I think that that created an extra step for us.*

Patients’ reluctance to use the app was mostly due to technological challenges, data privacy concerns, and a preference for phone calls over texting. Dentists suggested integrating additional features into the app, such as an in-system camera for patients to upload pictures and videos of the procedural site, integration with the EHR system, and including postoperative expectations and instructions in the platform for the patient to access after the procedure.

## Discussion

### Principal Findings

In this prospective, randomized, parallel-arm clinical trial evaluating the impact of an mHealth app on overall dental postoperative acute pain experience, we found no significant differences in pain experience or use of analgesic medication after painful dental procedures between the intervention (mHealth) and control (standard care) arms. Providers and patients, however, reported that the use of an mHealth platform had significant potential for improving patient-provider communication, patient-provider relationships, postoperative complication management, and the ability to customize pain medication prescribing. Almost all previously completed trials on the use of mobile technology apps for pain management in health care have focused on chronic pain. No trials have been reported in dentistry.

Several factors may have influenced the observed lack of difference between the study arms. First, comparisons between intervention and control groups were made on day 7. Previous studies suggest that postoperative dental pain is usually of short duration, reaching its maximum intensity in the early postoperative period (day 1) and petering out before day 7, regardless of the pain control technique [27]. Second, the included sites already have robust processes in place for postoperative patient care management (usual care). This includes 24-hour on-call dentists who are available by phone after standard hours for all questions and emergencies. This reduces the likelihood that patients in either arm would have experienced significant postoperative pain management issues. For the same reason, patients may have felt adequately involved in postoperative care decisions—hence, no difference was observed in patient satisfaction. Third, although there were four different types of “painful procedures” included in this study, the intervention arm seemed to have a lopsided proportion (681/851, 80% vs 359/674, 53.3% in the control arm) of patients undergoing the most painful procedures (oral surgery procedures). This uneven distribution resulted from the fact that patients assumed the randomization group of their providers. Fourth, the patient response rate decreased from day 1 to day 7 in the intervention group, with fewer patients responding to the surveys over time. This is a common issue with survey-based research, as patients may decline to respond to surveys, which could have introduced a selection bias. Finally, the importance of the design characteristics of mHealth apps should also be considered [28]. Characteristics such as reminders, notifications, incentives, follow-up, and the way these functions are provided can affect whether and how an app is used. It should also be noted that the timing and frequency of reminders must be well designed or they will be ignored by users [29]. A previous study found that users were most likely to use an app in 24 hours when the notification was sent at noon on weekends [30]. Gamification and incentive mechanisms such as virtual badges, unlocked levels, and behavior data comparison with other users are also considered driving forces for use [28].

Nevertheless, this study highlights the potential of PROs for providing valuable data for optimizing the delivery of care. Mobile phones have been shown to be an effective platform for assessing various aspects of patient health, including symptoms, symptom burden, health status, health behaviors, and health-related quality of life. In dentistry, mobile apps have been used to encourage evidence-based oral hygiene routines [31-34], triage emergencies [35], and prevention of dental caries [36]. Additionally, extensive evidence from systematic reviews and meta-analyses in medicine has demonstrated that mobile apps can effectively improve physical and mental health [37], medication adherence [16], and self-management of disease [17].

Our qualitative analysis revealed that the use of mHealth systems could be clinically useful in ways that have also been reported in other studies. For example, a study conducted in rural Ghana found that providers perceived the use of mHealth technology to be an approach to increasing health care access [38]. Similarly, another study reported that the use of mHealth technology improved patient communication [39]. In our study, providers perceived the mHealth app to be useful in guiding medication prescription, in contradiction to another study in which providers were concerned about overprescribing medication when using mHealth technology [40].

Postoperative pain measurement by recall is difficult to accurately determine. Research on autobiographical memory [41] indicates that recall is not just subject to random error but also is fraught with systematic bias, which can distort recall even after relatively short intervals. Many experiences are not retained in memory, so often the information we are asked to provide simply is not available for direct retrieval. A dramatic demonstration of the biases in recall—and an indication of how quickly these biases can set in—was reported by Redelmeier et al [42]. Summary ratings of pain by patients who had undergone a colonoscopy 20-30 minutes earlier were found to be unduly influenced by the peak level of pain (presumably because it was most salient) and the pain intensity at the end of the procedure (most recent). In other words, recall did not accurately represent the average pain over the interval because it was based on a few of the most memorable moments, essentially ignoring most of the experience. This shows the potential for bias even over short intervals. Besides being distorted by the operation of heuristic recall strategies, memory is also influenced by what we know and believe rather than actual recall. People unconsciously reorganize their “memories” to make them fit a coherent script or theory of events, or to reconcile events with what transpired subsequently [43]. Ecological momentary assessment (EMA) methods and technologies, designed to support the self-report of experience in the moment of daily life, are being considered poised to revolutionize human-centered research [44]. mHealth platforms could potentially be deployed more effectively if used in the context of EMA methods in which patients report their pain experience at the moment they are experiencing it and do not have to wait to receive survey prompts [45].

### Limitations

This study was conducted at two sites where standard postoperative care is exemplary, with disciplined adherence to evidence-based guidelines. Future studies should focus on pragmatic trials including sites that are more similar to everyday dental clinics with less stringent protocols, processes, or guidelines in place. EMA approaches should also be incorporated. As the primary outcome was pain intensity, a more predictable pain model, such as one limited to impacted third molar surgeries, might have been, in hindsight, better suited for this study.

### Conclusion

The study showed that using the mHealth platform did not have a significant impact on acute postoperative pain experience. However, patients and providers indicated increased improvements in patient-provider communication, patient-provider relationship, postoperative complication management, and the ability to manage pain medication prescribing.

## Supplementary material

10.2196/49677 Multimedia Appendix 1 Descriptive analysis for the Unified Theory of Acceptance and Use of Technology questionnaire.

10.2196/49677 Multimedia Appendix 2 Thematic analysis: barriers and facilitators.

10.2196/49677 Checklist 1 CONSORT-eHEALTH checklist (V 1.6.1).

## Abbreviations

APS-POQ-R: Revised American Pain Society Patient Outcome Questionnaire
EHR: electronic health record
EMA: ecological momentary assessment
mHealth: mobile health
PRO: patient-reported outcome
PROMIS: Patient-Reported Outcomes Measurement Information System
UTAUT: Unified Theory of Acceptance and Use of Technology
