# Supplementary material for: Evaluating the Impact of an mHealth Platform for Managing Acute Postoperative Dental Pain: Randomized Controlled Trial
Source: JMIR Mhealth Uhealth. 2023 Oct 20;11:e49677. doi: 10.2196/49677 (PMC10644946; doi:10.2196/49677)
Supplement: Multimedia Appendix 1 [file mhealth-v11-e49677-s001.pdf]

Please express your agreement with the statements below:								
1	2	3	4	5	6	7		
Strongly disagree	Disagree	Somewhat disagree	Neutral	Somewhat agree	Agree	Strongly agree		

**Performance expectancy Questionnaire Item**	**Median**	**Mean**	**SD**
PE1: I find FollowApp useful in my job.	6.0	5.4	1.5
PE2: Using FollowApp enables me to accomplish tasks more quickly.	4.0	4.4	1.9
PE3: Using FollowApp increases my productivity.	4.0	4.2	1.7
PE4: Using FollowApp will increase my chances of getting a better performance review.	5.0	5.1	1.2

Effort expectancy Questionnaire Item	Median	Mean	SD
EE1: My interaction with FollowApp is clear and understandable.	6.0	5.9	1.1
EE2: It is easy for me to become skillful at using FollowApp.	5.5	5.5	1.2
EE3: FollowApp is easy to use.	6.0	5.7	1.2
EE4: Learning to operate FollowApp is easy for me.	6.0	5.6	1.3

Social influence Questionnaire Item	Median	Mean	SD
SI1: People who influence my behavior think that I should use FollowApp.	5.0	4.9	1.7
SI2: People who are important to me think that I should use FollowApp.	4.5	4.8	1.6
SI3: The clinical management has been helpful in the use of FollowApp.	5.5	5.3	1.3
SI4: In general, the organization has supported the use of FollowApp.	7.0	6.3	1.0

Facilitating conditions Questionnaire Item	Median	Mean	SD
FC1: I have the resources necessary to use FollowApp.	7.0	6.3	1.0
FC2: I have the knowledge necessary to use FollowApp.	7.0	6.3	1.1
FC3: FollowApp is not compatible with other systems I use.	4.0	3.6	2.3
FC4: A specific person (or group) is available for assistance with FollowApp difficulties.	5.5	6.1	1.0
