# Supplementary material for: Evaluating the Impact of an mHealth Platform for Managing Acute Postoperative Dental Pain: Randomized Controlled Trial
Source: JMIR Mhealth Uhealth. 2023 Oct 20;11:e49677. doi: 10.2196/49677 (PMC10644946; doi:10.2196/49677)
Supplement: Multimedia Appendix 2 [file mhealth-v11-e49677-s002.pdf]

Themes	***Description***	***Quotes***
**1. Provider facilitators to mHealth utilization**	**a. Guides medication prescription/opioid prescription:** Dentists liked/appreciated how the mHealth application helped guide medication prescription by keeping a tab on patient's symptoms and pain levels postoperatively.<br><br>**b. Direct communication channel on PRO platform/ chat feature as free text:** One of the facilitators for dentists to using an mHealth application was the chat feature of the mHealth application used in the study which was perceived as a direct communication channel between the dentist and the patients.<br><br>**c. Alerts/ Trigger system:** Dentists approved the Alerts/Trigger system which was unique to the mHealth application used in this study. Dentists liked how they got alerts only for patients who had symptoms like postsurgical bleeding or swelling or their pain level was above 7 out of 10.<br><br>**d. Positive impact on patient care:** Dentists mentioned how the mHealth application helped reassure patients which positively impacted patient care and improved the patient dentist relationship because of continuous communication facilitated by the platform.<br><br>**h. Useful for providers who prefer texting:** Dentists stated that mHealth would be useful for dentists who prefer texting than calling their patients.<br><br>**i. Patient acceptance:**<br><br>Dentist perceived patient acceptance of mHealth application utilization is also a facilitator for dentists as	*"It seemed like we were a lot more accessible. It would lower their anxiety or if they were scared that something was going on, they were able to get answers a lot quicker. I think most of the patients liked it from what I remember hearing."*<br><br>*AND/OR*<br><br>*"I think that that is good because there are a lot of people. So yeah, I think that that's one thing the app provided that we didn't have before is that there are a lot of people who don't speak up when they're having those kind of concerns, and we don't have the bandwidth or the capacity to reach out to every single patient and ask them how they're doing. And so the app doing that is great cause it gets those people that are more reserved or more shy about expressing their pain because they didn't want to be a complainer."*<br><br>*AND*<br><br>*"Most of them were after like say extractions, day or two later they're having swelling or pain. We would comment back and try and*

	the dentists would like to use an mHealth if their patients like using it/accept it.	*find out like, what kind of pain instead of having to call them and make them talk and stuff. So we were able to just communicate through the website, or the messages, or whatever."*

**2. Provider Barriers to mHealth utilization**	**<u>a. Lack of personal touch through mHealth text messaging:</u>** Few dentists mentioned that following up via mHealth text messages has the limitation of lack of personal touch which is usually attained through traditional follow-up methods like a phone call with patients.<br><br>**<u>b. Additional step for providers:</u>**<br><br>One of the dentists considered that using the mHealth platform added a new workflow in terms of logging into the platform and using it to communicate with their patients, another found that they needed to train/teach the patients to use the platform was an inconvenience and did not eliminate the need for a follow-up phone call.<br><br>**<u>c. Third party involvement:</u>** A couple of providers mentioned that they would prefer a more automated and direct way of communicating through text messages rather than signing up for a third party mHealth application to follow up with their patients post operatively.<br><br>**<u>d. Invasion of family time/personal life:</u>** One of the dentists mentioned that the mHealth application invaded their personal time as they received text messages and alerts after work hours.<br><br>**<u>e. Patient barriers to mHealth:</u>** Dentist perceived patient barriers were identified (mentioned below) which can be considered as influencing factors for dentists' reluctance to using mHealth applications.	*"actually sometimes it (using the platform) was just an additional step, because I had to respond on the app and then call the patient because they still needed to talk to me. I still needed to talk to them. I felt like texting them wasn't enough. You know? So, yeah, I think in that way it was probably good for data collection and everything, but I think that that created an extra step for us."*<br><br>AND/OR<br><br>*"So in the very beginning, it was super stressful for me because they (patients) would text me on my phone, on my personal cell phone on my days off, and I couldn't respond to them and I felt like it was an urgent message... So that was stressful for me, because I felt like I was being asked to do something that I couldn't do."*

**3. Patient acceptance to mHealth utilization**	During the interviews, the dentists recalled patient experiences of using the mHealth application and provided their perceptions of patient acceptance to mHealth applications.<br><br>a. **<u>Sense of better care through continuous communication:</u>** Dentists stated that the mHealth usage for following up post operatively helped provide patients with a sense of better care. mHealth provided with a channel of continuous communication which helped reassure the patients that their dentist was available to answer their queries, which reduced their post-operative anxiety and improved the provider patient relationship.<br><br>b. **<u>Preference for using mHealth platform and messaging for communicating with their dentist:</u>** Dentists mentioned that many of their patients found the mHealth platform to be a quick tool for communication. Patients preferred answering the questions related to dental pain through surveys sent via mHealth platform instead of making phone calls or waiting for their dentist to call back. They also preferred using the chat feature over calling the dentist for providing details about their postoperative symptoms and asking questions. Some patients who were worried about their concerns not being as significant, also preferred texting over calling in the interest of their dentist's time.<br><br>**<u>c. Appreciative of mHealth option to connect with provider:</u>**<br><br>*Dentists also mentioned that the patients were appreciative of the mHealth application because it provided them with more accessibility to their dentist. Patients liked that the mHealth platform gave them*	*" A lot of them were very receptive. I feel, when we remembered to tell them and sign them up, they were really receptive. I think none of them were wanting to say no. Essentially, if they had a direct line to the doctor and then could ask questions over text, that seemed to be pretty helpful for them."*<br><br>*AND/OR*<br><br>*"I think a lot more people responded to the text messages. Like if they had questions or concerns for the doctors, rather than if they were experiencing pain after, like post-op pain, they didn't feel like calling in. They're like, "oh, it's not that bad. So I'm not going to call in""*<br><br>*AND/OR*<br><br>"Not really. I think it's a really good tool and I think a lot of people really liked the fact that they didn't have to try and call us if they had just a simple question. They could just send us a comment and I think it went really well."

	another option to contact the dentist directly rather than calling the front desk clinic staff.	

**4. Patient hesitancy to mHealth utilization**	During the interviews, the dentists recalled instances when their patients were reluctant to use the mHealth application used in the study and provided their perceptions of patient barriers to mHealth applications.<br><br>**a. Hesitancy in technologically challenged age groups:** Dentists found that their patients belonging to older age groups were less likely to use the mHealth application as they were often technologically challenged as compared to young patients. Dentists mentioned other reasons to decline mHealth usage among older aged groups were lack of cell phone/smartphone usage, and preference to call than text.<br><br>**b. Hesitancy to use mHealth due to data privacy:** Few dentists talked about how their patients were reluctant to use mHealth due to data privacy concerns especially seen among older aged patients and mentioned that some patients were afraid of being sent spam messages.<br><br>**c. Patient preference for calling than texting:** A couple of dentists mentioned that patients who preferred calling than texting their dentists/dental office were less likely to use mHealth for communication.	*"I think a lot of patients that I proposed it to were a little older, but maybe somebody who's more familiar with technology or interested in technology, that maybe younger folks would be more comfortable using the app versus the traditional way of calling or texting or whatever."*

**5. Future utilization of mHealth platform for Post-Operative Dental Pain Management**	During the interviews, XX % of the dentists mentioned that they would like to continue using an mHealth application to follow up postoperatively after dental procedures due multiple reasons such as those mentioned below.<br><br>**a. Enables texting patients than calling:** Many dentists mentioned that with the mHealth application, dentists have an option to text their patients which provides a direct communication channel to answer patient queries without having to call them. Dentists anticipated that their fellow dentists who prefer texting were more likely to use an mHealth application such as the one used in the study.<br><br>**b. Reduces burden on staff:** Dentists mentioned that because the mHealth application sent out follow-up surveys periodically and notified the dentist through the alert system, it helped in reducing the burden on the clinical staff by eliminating the need for follow-up phone calls.<br><br>**c. Suggested features:**<br><br>**- In-system camera accessibility for adding post-operative photos and videos.**<br><br>**- Integration with EHR.**<br><br>**- Pain management guide/expectations.**<br><br>**- Monitoring of mHealth by front desk staff**<br><br>Dentists suggested some additional features like an in-system camera accessibility to allow their patients to click and upload pictures and videos of the procedural site, integration of the mHealth platform with their Electronic Health Record system for better access to patient information and record maintenance and including post-operative expectations and instructions in the platform for the patient to access after the procedure. Many dentists also mentioned that they would want to use the mHealth application in daily	*"I would love it. Having worked at the front desk too, I think that we're able to just respond to patients in a much more timely manner. Especially at our organization right now, we have an appointment center that's short staffed and people don't always have the time to call. I think just from a patient care standpoint, I'd love to have something like this."*<br><br>AND/OR<br><br>*"So maybe (if) the app was linked to their account already to where we just clicked on something saying Follow Up to it... it would just be a lot easier where it would just transfer all the information to it....if it was linked to the system to where it already took the patient's name and just basically said, yes, do the follow up kind of a thing. I feel like that would be really easy to just keep doing."*

	practice and a couple of dentists mentioned that they would like help from the front desk staff to monitor it.	
